# Lightweight Monocular Depth Estimation with Local Feature Enhancement Modules and Guided Data Augmentation

**DOI:** 10.3390/s26165306

**Published:** 2026-08-21

**Authors:** Jae-young Lee, Soon-kak Kwon

**Affiliations:** Department of Computer Software Engineering, Dong-eui University, Busan 47430, Republic of Korea; wodud4143@naver.com

**Keywords:** monocular depth estimation, lightweight model, real-time inference, embedded systems

## Abstract

Although research on lightweight monocular depth estimation models has been actively conducted, achieving real-time deployment on edge devices remains challenging due to limited computational resources and memory capacity. To address these limitations, we propose a lightweight model for self-supervised monocular depth estimation. We cut the iterations of each feature-extracting block nearly in half, while our proposed Asymmetric Dilated Convolution module and a StarNext module compensate for the reduced model capacity. Specifically, the Asymmetric Dilated Convolution module captures horizontal and vertical structural features through asymmetric kernels, and the StarNext module fuses multi-scale features via element-wise multiplication. In model training, random cropping and scaling are applied for inducing the model to focus on localized object features. Additionally, we introduce a Disparity-guided Cutout technique based on the pre-inferred disparity map to randomly mask adjacent pixels. Simulation results on the KITTI dataset demonstrate that the proposed model reduces the number of parameters and GFLOPs by approximately 50% and 58%, respectively, without significant degradation in depth estimation accuracy compared to the baseline Lite-Mono. Furthermore, inference benchmarks on the Jetson Orin Nano platform demonstrate speedups of approximately 46.0%, 46.1%, 46.3%, and 52.0% across the MaxN, 25 W, 15 W, and 7 W power modes, respectively.

## 1. Introduction

Depth information is an essential component in spatial awareness, including autonomous driving, intelligent robotics, and augmented reality (AR). Although sensor-based depth measurement methods using LiDAR, stereo cameras, and structured light provide high accuracy, they suffer from high financial costs and heavy power consumption. Such limitations restrict the deployment of sensor-based methods for on-device applications [1]. Moreover, their limited spatial resolution and susceptibility to measurement noise hinder the accurate representation of fine-scale structures [2,3].

Unlike conventional approaches for acquiring depth maps, monocular depth estimation (MDE) generates depth maps directly from color images. By eliminating the need for dedicated depth sensors, MDE provides a cost-effective alternative for depth map acquisition. Early MDE studies [4,5,6] relied on supervised learning with ground-truth (GT) depth annotations obtained from sensors. However, acquiring accurate depth annotations remains challenging due to the inherent limitations of depth sensors and the difficulty of obtaining reliable depth measurements [7]. To alleviate the reliance on sensor-derived GT depth annotations, self-supervised MDE methods have been proposed [8,9,10]. These methods leverage multi-view geometric cues from stereo images or temporally adjacent monocular frames, allowing depth estimation to be learned through cross-view photometric reconstruction rather than explicit GT depth supervision. Recently, transformer-based MDE models have substantially improved performance by effectively capturing global context [11,12]. Nevertheless, existing MDE approaches remain difficult to deploy on edge platforms due to their high computational complexity and power consumption. Although lightweight approaches [13] have been proposed to reduce the computational burden of MDE, real-time inference under practical edge-computing constraints remain limited [14]. In addition, the reduced representational capacity of lightweight MDE models can limit their ability to capture fine-scale structures and preserve detailed depth information [3,15].

We propose a lightweight MDE model based on Lite-Mono [13] for real-time inference on edge platforms. The number of block repetitions within the model is reduced to achieve a lightweight design. To enhance the local feature representation capabilities constrained by this modification, we integrate feature enhancement modules into the baseline model. Specifically, we introduce a StarNext module and an Asymmetric Dilated Convolutions (ADC) module. The StarNext module implicitly projects feature representations into a high-dimensional space through star operations, while the ADC module enhances feature representations along object boundaries via asymmetric kernel shapes. In addition to these structural improvements, we employ a novel training strategy that guides the model to focus more on object features rather than the background during model training. Random cropping and scaling are applied to focus on localized object features. Additionally, we introduce a Disparity-guided Cutout technique that utilizes the pre-inferred disparity map to locate near-range pixels and mask the adjacent regions around them, thereby enabling the model to better learn the object context.

The main contributions of the proposed method are as follows:

- We introduce ADC and StarNext modules to enhance feature representations. This approach compensates for the performance limitations inherent in the reduced model capacity.
- We employ a novel training strategy utilizing customized cropping-scaling and a pre-inferred Disparity-guided Cutout method. Through this approach, the model focuses predominantly on capturing richer object-level representations in training.

## 2. Related Works

Eigen et al. [4], who first applied deep learning to monocular depth estimation, proposed a two-stage architecture consisting of a Global Coarse-Scale Network for predicting the overall depth structure and a Local Fine-Scale Network for refining detailed boundaries and textures. They proposed a scale-invariant error loss function to facilitate stable learning of relative depth relationships rather than absolute distances, thereby alleviating the scale ambiguity problem. Laina et al. [5] improved depth estimation accuracy through a fully convolutional residual network. Fu et al. [6] reformulated depth estimation as an ordinal regression problem. However, supervised methods are inherently limited by the requirement to collect large-scale ground truth (GT) data prior to training.

Self-supervised learning approaches that train networks without GT depth data are categorized into stereo image-based and monocular video-based methods. Both paradigms learn to satisfy geometric consistency between images captured from different viewpoints and employ a photometric reprojection loss as the primary training signal, which minimizes the photometric discrepancy between the reconstructed image synthesized from one viewpoint to another and the actual image. Godard et al. [8] proposed a network that derives depth from rectified stereo image pairs without camera pose estimation by exploiting the fixed distance between the two cameras and focal length. They introduced a left–right consistency loss that enforces geometric consistency between bidirectionally predicted disparities, effectively suppressing artifacts at depth discontinuities and occluded regions. Zhou et al. [9] presented a framework that separately trains a depth network and a camera pose network. The approach estimates the six degrees of freedom relative pose between consecutive frames and reprojects adjacent frames onto the current frame, thereby learning depth in a self-supervised manner. However, this method exhibited training instability in the presence of dynamic objects or when the camera remains stationary. Godard et al. [10] proposed Monodepth2, which introduced a per-pixel minimum reprojection loss to address erroneous matching caused by occluded pixels and an auto-masking stationary pixels strategy to mitigate training instability arising from scenarios where the camera is stationary or objects move at the same velocity as the camera. They also applied multi-scale estimation to suppress the texture-copy artifact that occurs at low resolutions, thereby achieving depth estimation performance comparable to stereo-based methods using only monocular video. However, the existing self-supervised networks including Monodepth2 still struggle to achieve sufficient inference speed in embedded environments with limited GPU performance and power budgets [3,14].

Zhang et al. [13] proposed Lite-Mono, a lightweight self-supervised MDE network combining CNN and Transformer. Lite-mono introduced a Consecutive Dilated Convolution (CDC) module that extracts multi-scale local features through successive dilated convolutions with different dilation rates, and a Local-Global Feature Interaction (LGFI) module employing Cross-Covariance Attention (XCA), which computes attention along the channel dimension instead of the spatial dimension. In network training, a ResNet-18 [16] was employed to estimate camera poses from consecutive frames. Both the estimated poses and the predicted depth were utilized to calculate the reprojection loss against the next frame. However, practical limitations remain in that sufficient computational headroom for real-time inference cannot be secured in low-power embedded environments.

## 3. Proposed Method

### 3.1. Network Architecture

**Figure 1:**
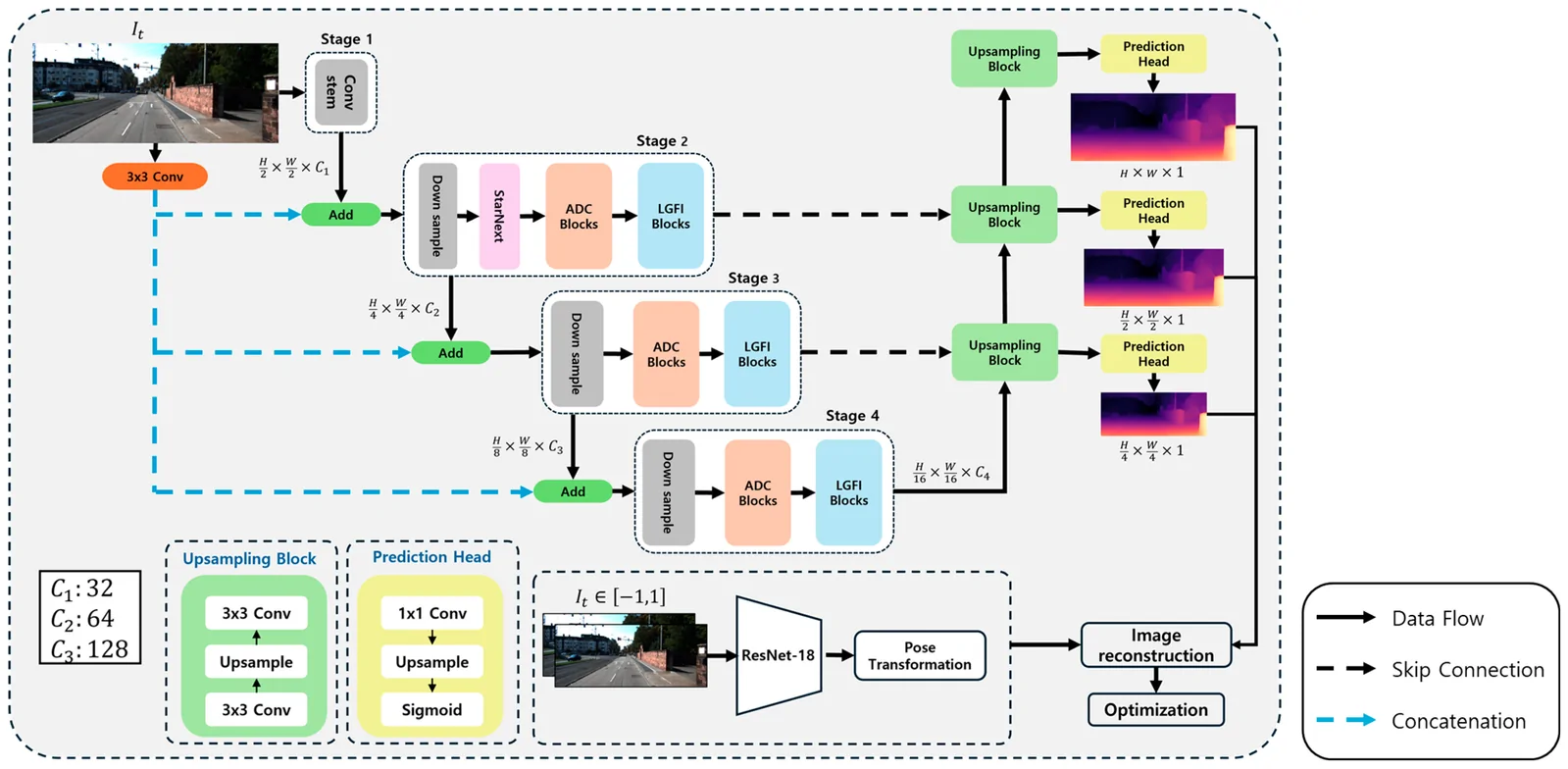
Architecture of the proposed network.

To achieve real-time capability in edge device environments, the network architecture of Lite-Mono is redesigned for lightweight efficiency, as shown in Table 1 and Figure 1. We decrease the frequency of convolution operations in the stem block. To compensate for the loss of spatial details caused by this reduction, we introduce a StarNext module into Stage 2 to enhance fine-scale structural features. Furthermore, we replace the conventional Consecutive Dilated Convolutions (CDC) blocks of Lite-Mono in Stages 2–4 with our Asymmetric Dilated Convolutions (ADC) blocks to enhance geometric directional information, while cutting the number of block repetitions nearly in half. To preserve spatial structural information more effectively during the downsampling process of the input shortcut, we replace the conventional average pooling of Lite-Mono with a 3 × 3 strided convolution. Average pooling inherently suffers from a loss of high-frequency spatial details, such as edges, corners, and textures [17,18] due to its inherent smoothing effect. In contrast, the strided convolution effectively retains essential spatial structural features [19]. To integrate the downscaled input image with the extracted features from the previous stages, we utilize element-wise addition rather than concatenation to maintain a compact channel dimension without additional computational overhead [20].

#### 3.1.1. StarNext Module

**Figure 2:**
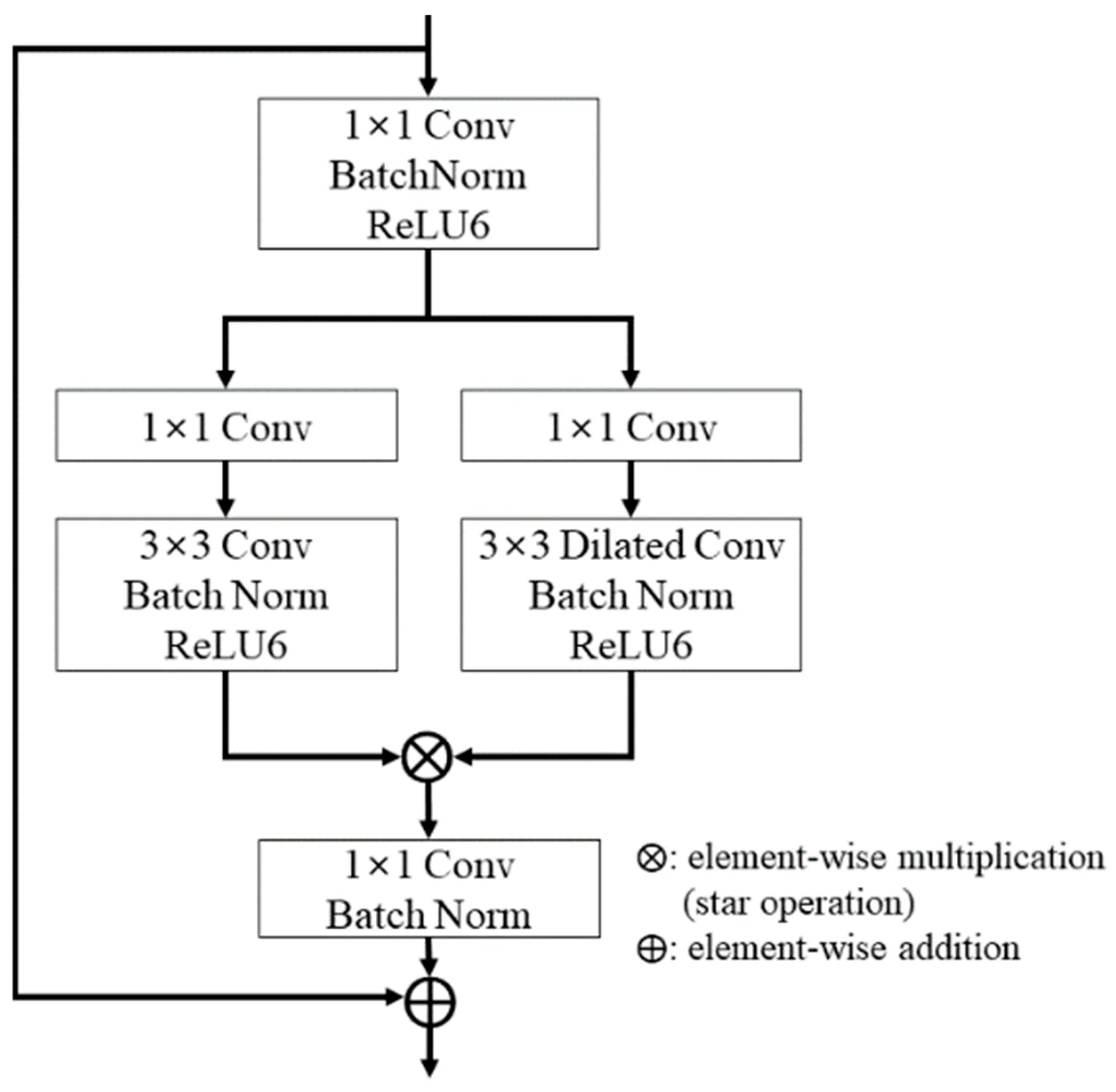
Structure of the StarNext module.

A StarNext module is introduced in Stage 2 to compensate for the loss of fine-scale structural features resulting from the reduction of the stem layer. The star operation, which represents the element-wise multiplication, is embedded in this module to generate all pairwise channel combinations of the two features, forming an implicit high-dimensional feature space analogous to a polynomial kernel, thereby achieving powerful feature representation [21]. ReLU6 is employed as the activation function to ensure hardware efficiency and suppress potential numerical explosions during the star operation. Figure 2 shows the structure of the proposed StarNext module.

The input feature $X_{sn}\in R^{H/2\times W/2\times C_{1}}$ is projected into a space with twice the channel dimension via a 1 × 1 convolution. The projected feature is fed into two branches. Both branches initially employ a 1 × 1 convolution to reduce the channel dimension by half, which mitigates the computational cost for the subsequent layers. The first branch employs a standard 3 × 3 convolution to capture local features. The second branch incorporates a 3 × 3 dilated convolution, thereby expanding the receptive field to aggregate global context without increasing computational overhead. The two features captured by the branches are integrated via star operation. Finally, the integrated feature is projected through a 1 × 1 convolution to align its representation and then aggregated with the input $X_{sn}$ via a residual skip connection. Table 2 describes the structural specifications of the StarNext module.

#### 3.1.2. Asymmetric Dilated Convolutions Module

**Figure 3:**
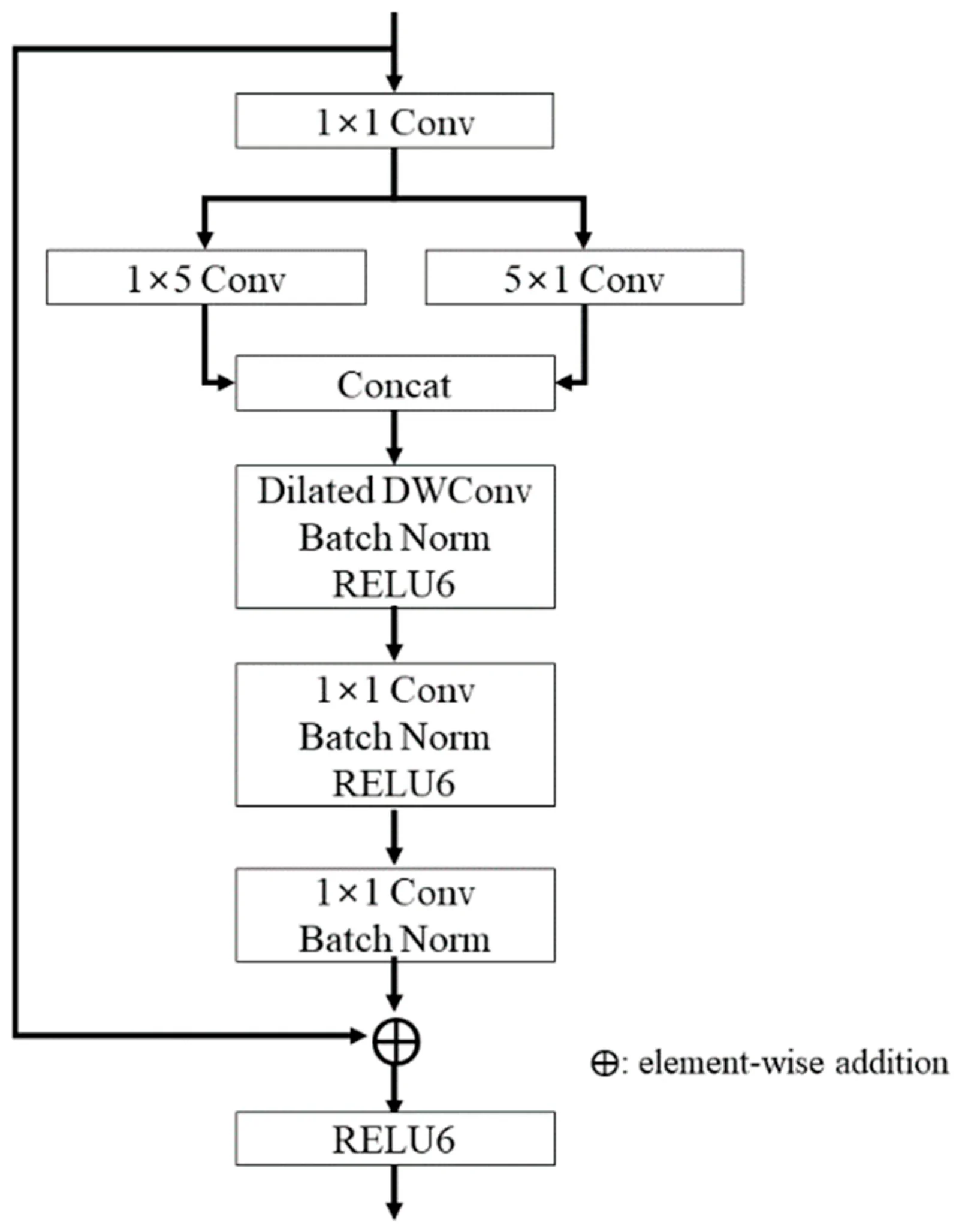
Structure of the Asymmetric Dilated Convolutions module.

We introduce an ADC module, which builds upon the CDC module by integrating an asymmetric operation to reinforce directional features. The asymmetric operation efficiently extracts features by factorizing a conventional *k* × *k* kernel into a 1 × *k* horizontal convolution and a *k* × 1 vertical convolution. Consequently, this decomposition strategy maintains the original receptive field while explicitly capturing horizontal and vertical structural cues, leading to enhanced directional expressiveness [22,23]. The overall architecture of the proposed ADC module is depicted in Figure 3.

For Stage *k*, the input feature $X_{ADC}\in R^{h_{k}\times w_{k}\times C_{n}}\;(n=1,2,3)$ passes through a 1 × 1 convolution. Subsequently, 1 × 5 and 5 × 1 asymmetric kernels extract their horizontal and vertical features, respectively. After concatenating the two features, a 3 × 3 dilated depthwise convolution and a pointwise convolution are applied to combine them with the channel-wise global spatial context, ultimately yielding rich local context. Subsequently, the channel dimension of this feature is reduced by half and then aggregated with the input $X_{ADC}$ via a residual skip connection. Table 3 describes the structural specifications of the ADC module.

### 3.2. Model Training Strategy

**Figure 4:**
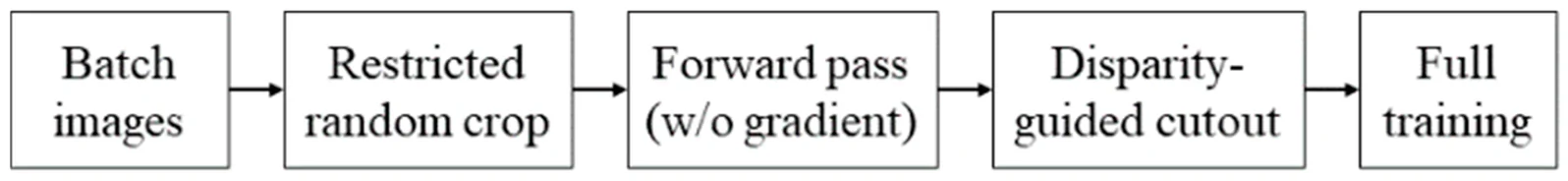
Model training flow per batch.

The restricted model capacity of the lightweight design may degrade the ability to extract the features of fine-scale structures [24,25]. To mitigate this degradation, we introduce two preprocessing strategies that enhance the representation of small-scale structures while preserving their essential shape and boundary information. For each batch, images are first transformed using Restricted Random Crop, which extracts randomly sized regions and rescales them. Subsequently, the model performs a preliminary forward pass without gradient computation to obtain guide disparity maps. The images are masked using Disparity-guided Cutout that randomly selects mask regions based on pixels with high disparity values. Figure 4 shows the proposed training pipeline.

#### 3.2.1. Restricted Random Crop

**Figure 5:**
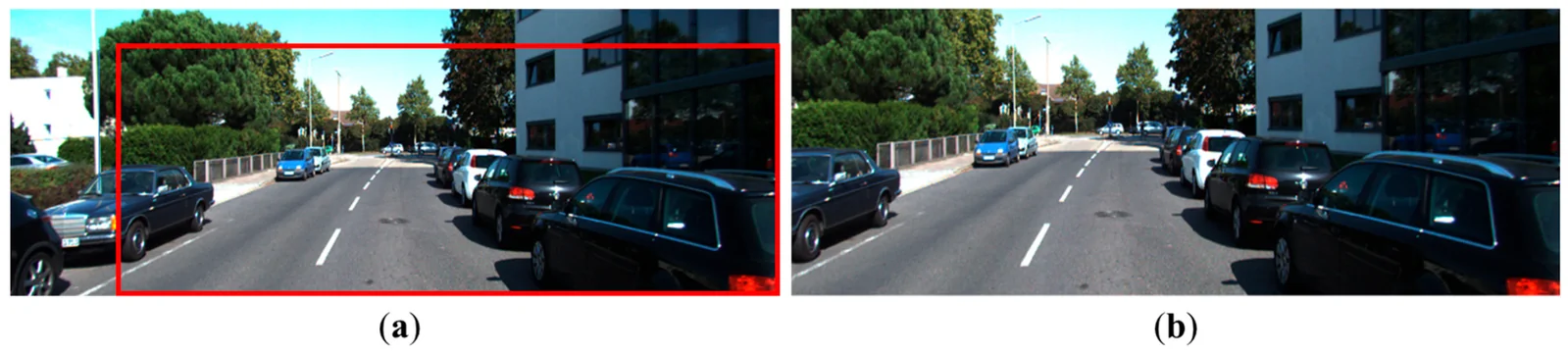
Applying Restricted Random Crop at 85% scale: (**a**) random region selection where the red box indicates the selected region; (**b**) image after cropping and scaling.

For guiding the model to focus more intensely on fine-scale structures, the images in each batch are randomly cropped and subsequently scaled to their initial resolution by bilinear interpolation. This enlarges the pixel representation of small-scale structures, whose features tend to gradually fade in deeper layers, for preserving essential shape and boundary information. The crop ratio is restricted to 85–95% due to the vulnerability of lightweight models to aggressive data augmentation [26,27]. Consistent with the baseline Lite-Mono augmentation pipeline, its cropping parameters are sampled only once for each training sample. The same crop parameters are then shared by the target frame and all source frames. Therefore, the target and source images undergo an identical geometric transformation. The camera intrinsic matrix is subsequently adjusted using the same crop offsets and resize factors, ensuring geometric consistency among the target frame, source frames, and camera intrinsics. Figure 5 illustrates that the proposed transformation expands the pixel footprint of object details.

#### 3.2.2. Disparity-Guided Cutout

**Figure 6:**
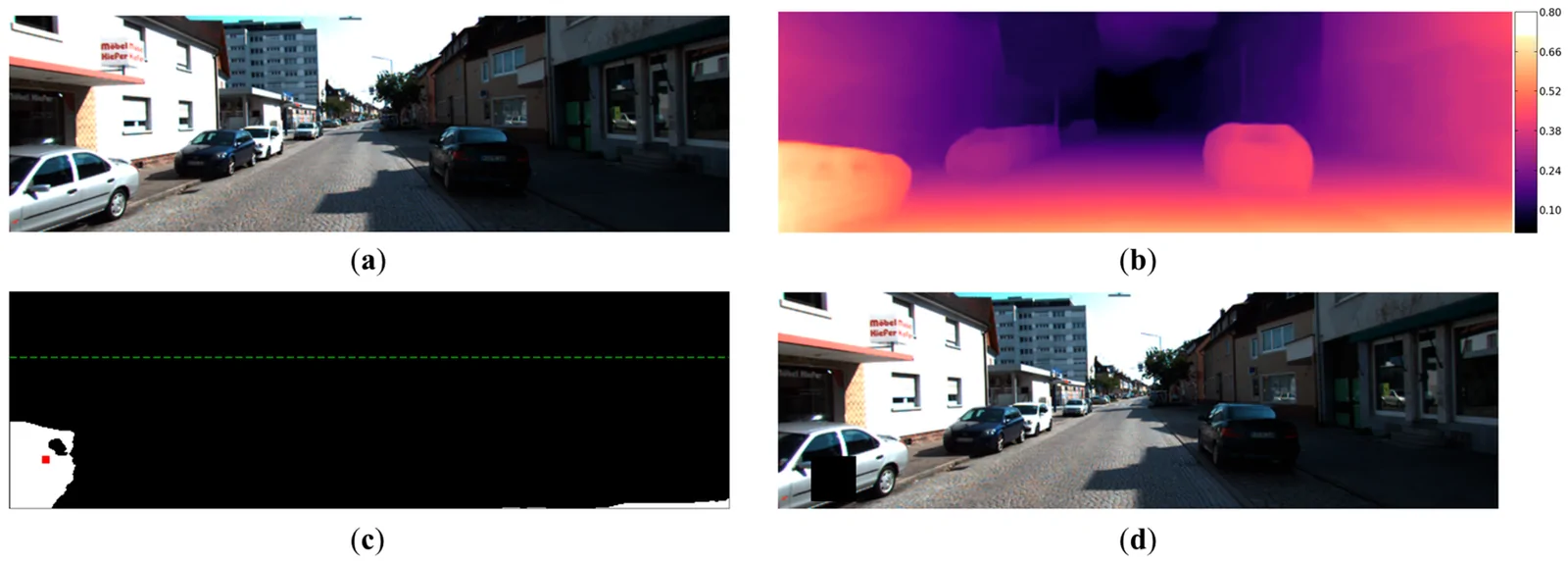
Image masking by Disparity-guided Cutout: (**a**) input image; (**b**) prior-guided disparity map; (**c**) candidate pixels for Disparity-guided Cutout; (**d**) masked image by Disparity-guided Cutout. In (**c**), the red square denotes a selected candidate pixel, and the green dashed line denotes the 30% height from the top.

Masking technique [28,29] has been used in model training to improve robustness and representation learning by encouraging the model to infer missing information from the remaining contextual cues. Cutout [28] is an image masking technique that randomly masks out square regions of images. However, Cutout could mask distant background regions in outdoor scenarios common to MDE tasks. Therefore, this indiscriminate masking limits its effectiveness in guiding structural learning. To enhance the learning efficiency of the MDE model, we introduce a Disparity-guided Cutout (DGC) technique that utilizes a preliminary disparity map to focus predominantly on foreground local regions. Prior to processing each batch, the disparity map is inferred by the model without further training. Then, pixel candidates for each image are designated based on disparity values within the top 1%. This exploration is restricted to the image domain excluding the upper 30% since the upper region tends to exhibit non-informative contexts in outdoor scenarios, such as the sky or distant backgrounds. Subsequently, zero-masking is applied to a square region centered around a randomly chosen candidate with its side length randomly determined within the range of 25 to 50 pixels. This encourages the model to learn richer contextual representations of foreground regions by reconstructing the masked region from its surrounding structural cues. As a result, the model is encouraged to learn structural patterns around foreground regions more actively. The DGC is applied exclusively to the target frame used as input to the depth estimation network, while the adjacent source frames remain unmasked. For pose estimation and photometric reprojection, separate unmasked copies of the target frame are retained and used throughout the geometric reconstruction process. Therefore, the DGC affects only the depth prediction input and does not alter the temporal correspondence between frames or directly modify the photometric reconstruction objective. To prevent unreliable disparity predictions at the beginning of training, the DGC is disabled during the first *e_warmup_* epochs and its application probability is then gradually increased over the subsequent *e_ramp_* epochs. The application probability of DGC at epoch *e_cur_* is as follows:

(1) $$p_{cutout}=\left\{\begin{array}{ll} 0, & e_{cur}\leq e_{warmup}\\ p_{tgt}\times \frac{e_{cur}-e_{warmup}}{e_{ramp}}, & e_{warmup}<e_{cur}\leq e_{warmup}+e_{ramp}\;\\ p_{tgt}, & otherwise \end{array}\right.\;,$$

where *p_tgt_* is the target DGC application probability. In this study, *p_tgt_*, *e_warmup_* and *e_ramp_* are set to 0.5, 3, and 5, respectively. Figure 6 illustrates the masking process of the proposed DGC.

#### 3.2.3. Loss Function

The proposed model is trained using the same loss functions as Lite-Mono. The photometric reprojection loss is computed as follows:

(2) $$L_{r}=\rho \left(I_{t},\hat{I_{t}}\right)\;,$$

where $I_{t}$ and ${\hat{I}}_{t}$ are the target input image and its reconstructed image, respectively, and $\rho \left(\cdot \right)$ is a photometric error between them. Following Lite-Mono, the photometric error is computed using a combination of the L1 Loss and the structural similarity index measure (SSIM). The reconstructed image $\left({\hat{I}}_{t}\right)$ is synthesized from a source image ($I_{t^{*}}$), where $t^{*}\in \left\{t-1,\;t+1\right\}$, using the relative camera pose estimated by PoseNet, the predicted depth of the target image, and the camera intrinsics. The photometric reprojection loss is computed by taking the minimum photometric error at each pixel across the two temporally adjacent source frames. The camera intrinsics are adjusted according to the image region retained after Restricted Random Crop. The unmasked target and source images without the DGC are used for photometric reconstruction to preserve the original inter-frame correspondences. The edge-aware smoothness loss is calculated as follows:

(3) $$\begin{array}{c} L_{s}=\left|\partial_{x}d_{t}^{*}\right|e^{-\left|\partial_{x}I_{t}\right|}+\left|\partial_{y}d_{t}^{*}\right|e^{-\left|\partial_{y}I_{t}\right|}\\ d_{t}^{*}=d_{t}/\bar{d_{t}} \end{array}\;,$$

where *d_t_* and $\bar{d_{t}}$ are the predicted disparity map and its spatial mean, respectively. $d_{t}^{*}$ is the mean-normalized inverse depth. The total loss is as follows:

(4) $$\begin{array}{c} L_{total}=L_{r}+\lambda_{s}L_{s} \end{array},$$

where $\lambda_{s}$ is a weight of the smoothness loss.

## 4. Experiment Results

### 4.1. Experimental Settings

We validated the proposed model on the KITTI dataset [30]. The Eigen split [4] was applied to the dataset to construct the training, validation, and test sets, which consist of 38,383, 4267, and 697 images, respectively. The training environment comprised an Ubuntu 20.04 operating system, an NVIDIA GeForce RTX 4090 GPU, and 32 GB of memory. Additionally, inference speed experiments were conducted separately on the Jetson Orin Nano 8GB platform to verify the practical effectiveness in low-power embedded environments. The proposed model and training pipeline were implemented using Python 3.11 and PyTorch 2.8.0. Identical hyperparameter settings were applied across all experiments. Table 4 shows the hyperparameters used for training.

### 4.2. Metrics

For quantitative evaluation of depth estimation performance, we adopt seven metrics that are standard in the monocular depth estimation field. The error metrics include Absolute Relative Error (AbsRel), Squared Relative Error (SqRel), Root Mean Squared Error (RMSE), and Root Mean Squared Logarithmic Error (RMSLE).

(5) $$AbsRel=\frac{1}{N}\sum_{i=1}^{N}\frac{\left|{\hat{d}}_{i}-d_{i}\right|}{d_{i}}\;,$$

(6) $$SqRel=\frac{1}{N}\sum_{i=1}^{N}\frac{{\left({\hat{d}}_{i}-d_{i}\right)}^{2}}{d_{i}}\;,$$

(7) $$RMSE=\sqrt{\frac{1}{N}\sum_{i=1}^{N}{\left({\hat{d}}_{i}-d_{i}\right)}^{2}},$$

(8) $$RMSLE=\sqrt{\frac{1}{N}\sum_{i=1}^{N}{\left(\log{\hat{d}}_{i}-\log d_{i}\right)}^{2}}\;,$$

where $d_{i}$ and ${\hat{d}}_{i}$ are GT and predicted depths, respectively, and *N* is the number of pixels. SqRel is employed as the primary metric in this paper for evaluating depth estimation accuracy for near-range objects. SqRel has a structure that squares the error and normalizes it by the ground truth depth value. For near-range pixels with small depth values, the denominator becomes smaller, causing even errors of the same magnitude to be reflected more significantly in the metric, and the squared error term makes the metric more sensitive to large errors. Due to these mathematical properties, SqRel can measure depth estimation errors in near-range regions more sensitively than other metrics, making it a suitable evaluation criterion for quantitatively verifying the effectiveness of Restricted Random Crop and DGC, both of which aim to enhance learning for near-range objects. In addition, we adopt the threshold-based ratios *δ^i^* (*i* = 1, 2, 3) as accuracy metrics. The *δ^i^* indicates the proportion of pixels satisfying *δ* < 1.25*^i^*, where *δ* for each pixel is defined as follows:

(9) $$\delta =\max\left(\frac{{\hat{d}}_{i}}{d_{i}},\frac{d_{i}}{{\hat{d}}_{i}}\right)\;.$$

### 4.3. Results

We evaluate and compare the performance between the proposed method and the baseline Lite-Mono [13]. Table 5 presents the performance comparison results on the 697 test images of the KITTI test set. The comparison reveals that Lite-Mono holds a marginal advantage in the SqRel and RMSE metrics; however, the differences are negligible. Conversely, the proposed method achieves an improved AbsRel value and attains 0.871 in the accuracy metric *δ*^1^, maintaining a level of performance comparable to Lite-Mono on the KITTI dataset. The in-domain evaluation on KITTI indicates that the proposed lightweight design results in only negligible degradation in depth estimation accuracy compared with the baseline.

Table 6 compares the depth estimation accuracy of the proposed and baseline models across different depth ranges on the KITTI test set. For the distance-wise evaluation, pixels were grouped into depth intervals according to their reference depths, and the prediction errors were independently evaluated for each interval. For depths below 60, the proposed model achieved accuracy metrics comparable to those of the baseline model. However, a noticeable performance degradation was observed in the 60–80 depth interval. This result suggests that the introduced modules and training strategies are more effective in preserving or enhancing feature representations for near-range objects, whereas their benefits become relatively limited for distant regions. Consequently, in the far-range interval, the reduced model capacity resulting from the lightweight network configuration becomes more pronounced, leading to a larger performance degradation.

To evaluate the generalization capability of the proposed model, cross-dataset experiments were conducted on the Make3D dataset. Make3D is an outdoor monocular depth estimation dataset comprising 400 training samples and 134 test samples. The RGB images have a resolution of 2272 × 1704 pixels, while the corresponding ground-truth depth maps have a resolution of 305 × 55 pixels. In this experiment, only the 134 test images were used, and both the baseline Lite-Mono and the proposed model trained exclusively on KITTI were directly evaluated on Make3D without additional training or fine-tuning. As shown in Table 7, the proposed model exhibits a moderate degradation in depth estimation accuracy compared with the baseline Lite-Mono, with the performance difference being relatively pronounced in SqRel. This performance degradation suggests that the reduced model capacity resulting from the decreased number of parameters may limit the model’s ability to learn representations that generalize across datasets.

An ablation study was conducted to quantitatively analyze the performance contribution of each proposed component. The experiments comprise the model with all proposed methods applied, models with each component removed individually, and models with each component applied in isolation, along with comparisons based on the presence or absence of Restricted Random Crop. Where the ADC module is excluded, the CDC module from the baseline Lite-Mono is employed instead. Table 8 presents the results of the ablation study. The results show that applying Restricted Random Crop yields marginal metric fluctuations in some configurations, but overall performance improves across most metrics. In particular, an improvement of approximately 5.9% for the full model is observed in the SqRel metric compared to the model without Restricted Random. This implies that Restricted Random Crop helps the model learn more discriminative representations of foreground structures. Furthermore, the full model consistently outperforms its partial variants, indicating that the proposed components provide complementary benefits.

To validate the effectiveness of the proposed DGC, we conduct a comparative experiment against Bottom-Half Random Cutout, which randomly selects masking regions from the bottom 50% of the input image. Table 9 presents the performance comparison results between the two strategies. The results show that DGC records lower values than Bottom-Half Random Cutout across most error metrics, including AbsRel, SqRel, and RMSLE, and also achieves higher values in the accuracy metrics *δ*^1^ and *δ*^2^. In particular, an improvement of approximately 3.4% is observed in SqRel, from 0.882 to 0.852, confirming that adaptive masking based on the disparity map is effective in improving near-range depth estimation accuracy. However, the proposed DGC has higher RMSE than the bottom-half random cutout. These results suggest that the DGC improves the overall relative depth estimation accuracy by strengthening feature representations particularly for nearby regions, while relatively large absolute errors may remain in a limited subset of pixels, such as those in distant regions, resulting in the increased RMSE.

The inference speed was evaluated on the Jetson Orin Nano 8GB platform (NVIDIA Corporation, Santa Clara, CA, USA) to verify the real-time inference capability of the proposed method. Jetson_clocks, an NVIDIA-provided utility for controlling hardware clock frequencies on Jetson platforms, was enabled to fix the CPU, GPU, memory, and EMC clocks at their maximum operating frequencies. The baseline Lite-Mono model and the proposed model were exported to ONNX using operator set version 17, which specifies the ONNX operators used to represent the model architecture. Then, they were converted into a TensorRT engine using TensorRT 10.3.0 with FP16 precision mode enabled. To comprehensively evaluate the practicality in low-power embedded environments, experiments are conducted across four power modes: MaxN, 25 W, 15 W, and 7 W. For each power mode, inference was repeated 1000 times after 500 warm-up inference iterations, and mean and standard deviation of the inference time were computed. Table 10 compares the number of parameters, GFLOPs, and average inference time per power mode for each model. The results show that the proposed method reduces the number of parameters by approximately 50% and GFLOPs by approximately 58% compared to Lite-Mono. In terms of inference speed, speedups of approximately 46.0%, 46.1%, and 46.3% are achieved under the MaxN Super, 25 W, and 15 W modes, respectively. Notably, under the most power-constrained 7 W mode, Lite-Mono requires 36.613 ms, exceeding the real-time processing threshold of 33.3 ms (30 FPS), whereas the proposed method achieves 17.583 ms, satisfying the real-time inference requirement with an approximately 52% speedup. This indicates that the real-time advantage of the proposed method becomes more pronounced as the power constraint increases and demonstrates the applicability in practical embedded application environments where auxiliary AI modules or additional algorithms must operate concurrently alongside the depth estimation model.

Table 11 compares the results obtained using different channel configurations of the network. When the channel configuration was changed from (32, 64, 128) to (30, 60, 120), the depth estimation performance metrics showed degradation. Despite its lower computational complexity, the model with channel dimensions of (30, 60, 120) exhibited a slightly longer inference time after TensorRT optimization than that with (32, 64, 128). This behavior can be attributed to hardware-aware execution characteristics. In FP16 inference, Tensor Core operations favor appropriately aligned tensor dimensions, whereas channel dimensions such as 30 and 60 are not aligned to multiples of eight. Consequently, the reduced arithmetic complexity does not necessarily translate into lower execution latency because TensorRT may employ padding, tensor reformatting, or different execution tactics for these channel configurations [32,33,34,35].

Table 12 compares the performance of recently reported lightweight MDE models on the KITTI dataset. For a fair comparison, only results evaluated at the same input resolution of 640 × 192 were considered. The proposed model exhibits the lowest reported GFLOPs. Although a direct comparison is difficult because the GFLOP measurement protocols may differ across studies, this result suggests that reducing the number of repeated blocks at each stage and decreasing the channel dimensions can contribute to lowering the computational complexity of the proposed model. Despite these architectural simplifications, the proposed StarNext and ADC modules appear to compensate for the reduced model capacity, thereby limiting the degradation in depth estimation performance.

**Figure 7:**
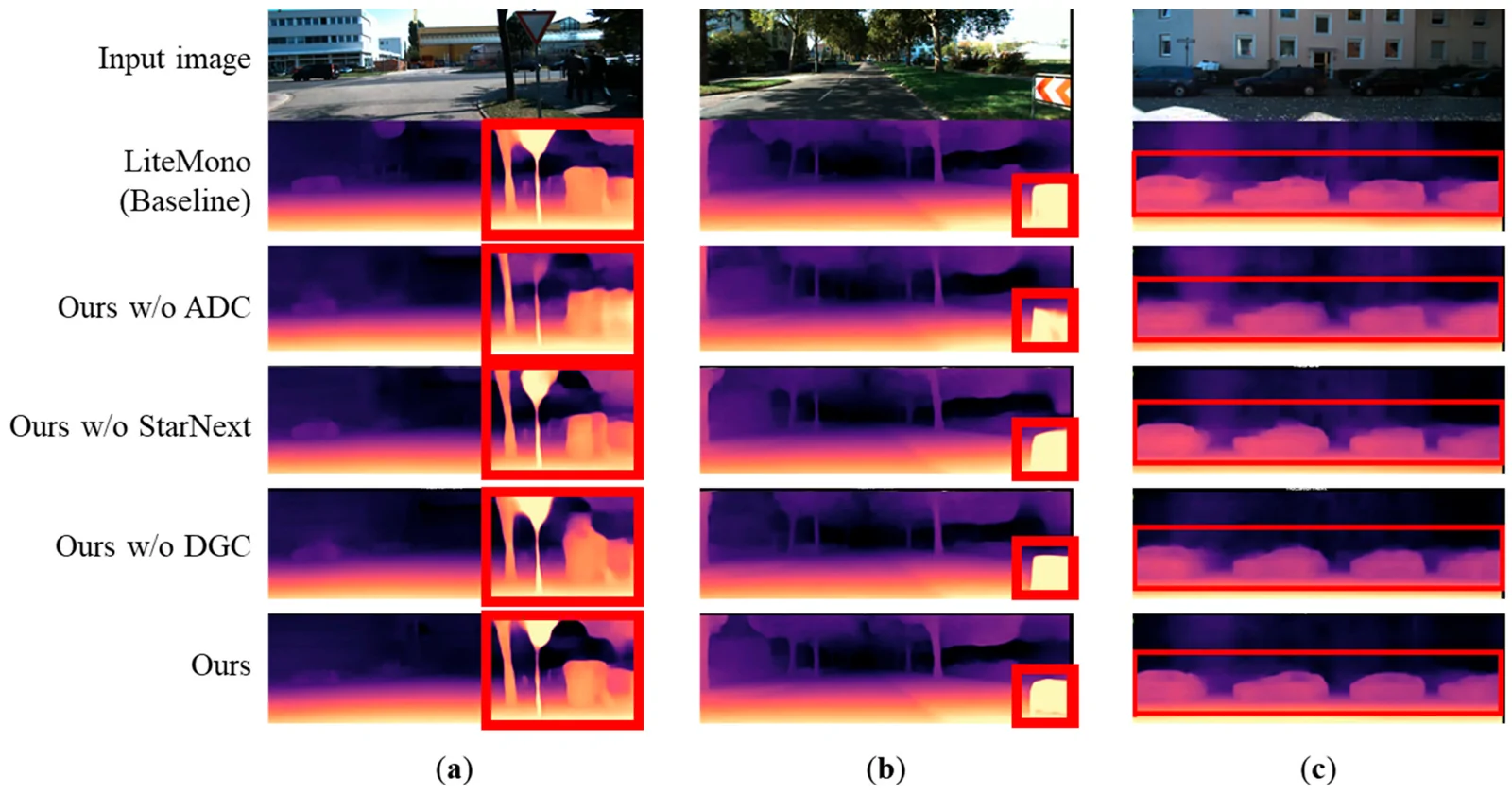
Comparison of depth estimation results: (**a**) for thin objects; (**b**) for single near-range object; (**c**) for multiple adjacent objects. The red boxes indicate regions containing near-range objects. Brighter pixel colors indicate relatively smaller depth values.

Figure 7 presents qualitative depth estimation comparison results across three scenarios. As shown in Figure 7, the visual comparisons are displayed from top to bottom in the following order: the input image, the baseline Lite-Mono, the proposed model excluding the ADC module, excluding the StarNext module, excluding the DGC strategy, and the full model. Figure 7a–c corresponds to depth estimation results for thin objects, near-range object, and multiple adjacent objects, respectively. In Figure 7a, the models without ADC and DGC have less distinct depth discontinuities between adjacent objects. Compared with these variants, the full model stably preserves the triangular shape while separating the gap between the sign and the background more clearly. In Figure 7b, the partial variants incorrectly estimate the empty background region beneath the near-range sign with a depth similar to that of the object region. In contrast, the full model preserves the overall shape of the near-range sign while representing the support structure more distinctly than the baseline model. In Figure 7c, the depth discontinuities of vehicles are insufficiently preserved in the models without the ADC module and the StarNext module. When the DGC is not applied, inconsistent depth estimation is observed in the planar regions within vehicles. Although the full model is slightly less effective than the baseline Lite-Mono in preserving depth details around the window regions, it largely maintains comparable depth discontinuity preservation at vehicle boundaries.

## 5. Conclusions

We proposed a method that redesigns the model architecture of Lite-Mono for real-time self-supervised monocular depth estimation in low-power embedded environments and compensates for the local feature representation degraded by the lightweight design. Specifically, we designed the ADC and StarNext modules for directional feature extraction based on asymmetric kernels and for implicit high-dimensional feature representation through the Star Operation, respectively. In addition, we introduced the Disparity-guided Cutout and Restricted Random Crop techniques to enhance feature representation learning. The experimental results on the KITTI dataset demonstrated that the proposed model reduced the number of parameters and GFLOPs by approximately 50% and 58%, respectively, compared to the baseline Lite-Mono, while mitigating the performance degradation caused by model lightweighting. The inference speed evaluation conducted on the Jetson Orin Nano 8GB demonstrated approximately 46–52% speedups across all power modes. The qualitative evaluation further suggests that the ADC module, StarNext module, and the introduced training strategy partially compensate for the reduction in representational capacity caused by the reduced channel dimensions, thereby mitigating the performance degradation associated with network lightweighting. However, the cross-dataset evaluation indicates that the proposed model exhibits weaker generalization performance than the baseline model, suggesting that further improvements are required to enhance its robustness to unseen domains. In future work, we plan to introduce Consistency Regularization [41], which enforces depth consistency between outputs obtained by applying different augmentations to the same input, to improve the depth estimation stability of the lightweight model without additional parameters. Additionally, we intend to design a lightweight global context learning module based on Linear Attention [42] to enhance global representational capacity while maintaining computational efficiency.

## Figures and Tables

**Table 1:** Comparison of network structure between baseline Lite-Mono and the proposed model. *S* denotes stride, underlined items indicate modules or settings that were either added to or replaced in the baseline model, and “–” indicates no corresponding module in the respective network.

Stage	Output Size	Structure	
		Baseline (Lite-Mono)	Proposed
Stage 1 (Stem)	H/2 × W/2 × *C*_1_	3 × 3 Conv (*S* = 2)	3 × 3 Conv (*S* = 2)
		3 × 3 Conv × 2	–
Stage 2	H/4 × W/4 × *C*_2_	Downsampling	Downsampling
		–	StarNext block
		CDC blocks × 3	ADC blocks × 3
		LGFI block	LGFI block
Stage 3	H/8 × W/8 × *C*_2_	Downsampling	Downsampling
		CDC blocks × 3	ADC blocks × 2
		LGFI block	LGFI block
Stage 4	H/16 × W/16 × *C*_3_	Downsampling	Downsampling
		CDC blocks × 9	ADC blocks × 3
		LGFI block	LGFI block
Input shortcut		Avg Pooling	Strided Conv
		Concat	Element-wise add
*C*_1_		48	32
*C*_2_		80	64
*C*_3_		128	128
Parms. (M)		3.069	1.520

**Table 2:** Structural specifications of the StarNext module. *D* denotes dilation rate.

Layer	Output Channels	Structure
Input	*C*_1_	–
Projection 1	*2C*_1_	1 × 1 Conv
		Batch Norm
		ReLU6
Branch1	*C*_1_	1 × 1 Conv
		3 × 3 Conv
		Batch Norm
		ReLU6
Branch2	*C*_1_	1 × 1 Conv
		3 × 3 Conv (*D* = 2)
		Batch Norm
		ReLU6
Star operation	*C*_1_	Element-wise multiplication
Projection 2	*C*_1_	1 × 1 Conv
		Batch Norm
Skip connection	*C*_1_	Add

**Table 3:** Structural specifications of the ADC module. *D* denotes dilation rate.

Layer	Output Channels	Structure
Input	*C_n_*	–
Projection 1	*C_n_*	1 × 1 Conv
Horizontal branch	*C_n_*	1 × 5 Conv
Vertical branch	*C_n_*	5 × 1 Conv
Fusion	2*C_n_*	Concat
Dilated depthwise Conv	2*C_n_*	3 × 3 Conv
		(*D* = [1, 2] in Stage 2–3, [2, 3, 5] in Stage 4)
		Batch Norm
		ReLU6
Pointwise Conv	*C_n_*	1 × 1 Conv
		Batch Norm
		ReLU6
Projection 2	*C_n_*	1 × 1 Conv
		Batch Norm
Skip connection	*C_n_*	Add
		ReLU6

**Table 4:** Hyperparameter settings.

Hyperparameter			Value
Batch size			12
Epoch			100
Input size			640 × 192
Optimizer			AdamW
Scheduler			Cosine annealing schedule with warm restarts [31]
Pretrained			No
Random seed			42
Learning rates	Main network	Initial learning rate	5 × 10^−4^
		Minimum learning rate	5 × 10^−6^
		First cycle step size	35
	PoseNet	Initial learning rate	1 × 10^−4^
		Minimum learning rate	1 × 10^−5^
		First cycle step size	35

**Table 5:** Comparison of depth estimation accuracy with baseline Lite-Mono. The better values are highlighted in bold.

Model	AbsRel	SqRel	RMSE	RMSLE	*δ* ^1^	*δ* ^2^	*δ* ^3^
Baseline [13]	0.116	**0.837**	**4.740**	**0.194**	**0.871**	**0.958**	**0.981**
Proposed	**0.115**	0.852	4.752	0.194	0.871	0.958	0.980

**Table 6:** Comparison of depth estimation accuracy with baseline Lite-Mono across different depth ranges on the KITTI test set. The better values within each depth interval are highlighted in bold.

Depth Interval	Model	AbsRel	SqRel	RMSE	RMSLE	*δ* ^1^	*δ* ^2^	*δ* ^3^
[0, 10)	Baseline	0.087	0.332	1.171	**0.119**	**0.945**	**0.985**	**0.993**
	Proposed	**0.086**	**0.278**	**1.131**	**0.119**	0.939	**0.985**	**0.993**
[10, 20)	Baseline	**0.105**	0.666	2.696	**0.164**	**0.898**	**0.965**	**0.983**
	Proposed	**0.105**	**0.573**	**2.553**	**0.164**	0.896	**0.965**	**0.983**
[20, 40)	Baseline	0.167	1.932	6.905	0.269	0.760	**0.909**	**0.954**
	Proposed	**0.165**	**1.771**	**6.657**	**0.272**	**0.762**	**0.909**	0.951
[40, 60)	Baseline	**0.217**	3.834	12.793	**0.336**	**0.602**	**0.851**	**0.928**
	Proposed	**0.217**	**3.824**	**12.760**	0.345	0.593	0.843	0.922
[60, 80)	Baseline	**0.220**	**5.256**	**17.309**	**0.346**	**0.565**	**0.822**	**0.919**
	Proposed	0.244	6.345	18.965	0.388	0.502	0.773	0.895

**Table 7:** Cross-dataset performance comparison on the Make3D dataset. The better values are highlighted in bold.

Model	AbsRel	SqRel	RMSE	RMSLE	*δ* ^1^	*δ* ^2^	*δ* ^3^
Baseline [13]	**0.309**	**3.085**	**7.089**	**0.169**	**0.558**	**0.780**	**0.882**
Proposed	0.329	3.962	7.591	0.179	0.542	0.753	0.868

**Table 8:** Ablation study results. The best and second-best results are highlighted in bold and underline, respectively. For modules without checkmark, the corresponding module in baseline Lite-Mono is used.

ADC	StarNext	Disparity-Guide Cutout	Restricted Random Crop	AbsRel	SqRel	RMSE	RMSLE	*δ* ^1^	*δ* ^2^	*δ* ^3^
✓				0.125	0.944	4.880	0.200	0.856	0.953	0.980
	✓			0.124	0.927	4.930	0.202	0.854	0.952	0.979
✓	✓			0.122	0.959	5.018	0.200	0.861	0.953	0.979
		✓		0.124	0.905	4.917	0.200	0.856	0.953	**0.981**
✓		✓		0.127	0.948	5.002	0.204	0.852	0.951	0.978
	✓	✓		0.124	0.927	4.937	0.202	0.856	0.952	0.979
✓	✓	✓		0.121	0.905	4.904	0.199	0.860	0.954	0.980
✓			✓	0.120	0.894	4.852	0.199	0.861	0.954	0.979
	✓		✓	0.123	0.889	4.878	0.201	0.851	0.953	0.980
✓	✓		✓	0.122	0.909	4.964	0.202	0.857	0.952	0.979
		✓	✓	0.124	0.906	4.917	0.203	0.850	0.953	0.980
✓		✓	✓	0.120	0.923	4.914	0.198	0.863	0.954	0.979
	✓	✓	✓	0.127	0.928	5.031	0.204	0.845	0.950	0.979
✓	✓	✓	✓	**0.115**	**0.852**	**4.752**	**0.194**	**0.871**	**0.958**	0.980

**Table 9:** Comparison of Cutout Strategies. The better values are highlighted in bold.

Cutout Strategy	AbsRel	SqRel	RMSE	RMSLE	*δ* ^1^	*δ* ^2^	*δ* ^3^
Bottom-half random cutout	0.116	0.882	**4.485**	0.195	0.869	0.956	0.980
Disparity-guided cutout	**0.115**	**0.852**	4.752	**0.194**	**0.871**	**0.958**	**0.980**

**Table 10:** Comparison of computational complexity.

Model	Parms. (M)	GFLOPs	Inference Time (ms) (Standard Deviation)			
			MaxN	25 W	15 W	7 W
Baseline [13]	3.069	5.032	9.624 (0.993)	10.476 (1.300)	16.934 (4.176)	36.613 (5.675)
Proposed	1.520	2.079	5.196 (0.831)	5.652 (0.920)	9.098 (1.196)	17.583 (2.895)

**Table 11:** Comparison of model performance across different channel configurations.

(*C*_1_, *C*_2_, *C*_3_)	AbsRel	SqRel	RMSE	RMSLE	*δ* ^1^	*δ* ^2^	*δ* ^3^	Parms. (M)	GFLOPs	Inference Time (ms)			
										MaxN	25 W	15 W	7 W
(32, 64, 128)	0.115	0.852	4.752	0.194	0.871	0.958	0.980	1.520	2.079	5.196	5.652	9.098	17.583
(30, 60, 90)	0.119	0.905	4.815	0.197	0.866	0.956	0.979	1.338	1.835	5.801	6.168	10.534	18.052

**Table 12:** Performance comparison with recent lightweight monocular depth estimation models. Pretrain indicates whether the model was initialized with weights pretrained on an ImageNet dataset prior to MDE training. The best values are highlighted in bold.

Model	Year	Pretrain	AbsRel	SqRel	RMSE	RMSLE	*δ* ^1^	*δ* ^2^	*δ* ^3^	Parms. (M)	GFlops
Lite-Mono [13]	2023	Y	0.116	0.837	4.740	0.194	0.871	0.958	0.981	3.1	5.0
RTIA-Mono [36]	2025	N	0.118	0.858	4.810	0.196	0.865	0.955	0.980	1.4	3.7
MsGf [15]	2025	N	0.117	0.871	4.794	0.194	0.871	0.958	0.981	**0.8**	2.9
MonoLENS [37]	2025	Y	0.110	0.833	4.644	0.185	0.883	0.962	0.982	1.8	4.0
RTS-Mono-XS [38]	2025	Y	**0.101**	**0.690**	**4.357**	**0.175**	**0.897**	**0.966**	**0.984**	2.4	6.0
PuriLight [39]	2025	Y	0.106	0.747	4.536	0.182	0.890	0.964	**0.984**	2.7	7.1
XiDepth [40]	2026	N	0.120	0.941	4.876	0.198	0.870	0.956	0.980	**0.8**	3.0
Proposed	2026	N	0.115	0.852	4.752	0.194	0.871	0.958	0.980	1.5	**2.0**

## Data Availability

The data are contained within the article.
